# Role of STAT3 in pancreatic cancer

**DOI:** 10.37349/etat.2024.00202

**Published:** 2024-01-17

**Authors:** Zachary Hamel, Sierra Sanchez, David Standing, Shrikant Anant

**Affiliations:** Curtin University, Australia; Istituto Nazionale Tumori-IRCCS-Fondazione G. Pascale, Italy; Department of Cancer Biology, University of Kansas Medical Center, Kansas City, KS 66160, USA

**Keywords:** Signal transducer and activator of transcription, tumor microenvironment, pancreatic cancer

## Abstract

Pancreatic cancer remains a serious and deadly disease, impacting people globally. There remain prominent gaps in the current understanding of the disease, specifically regarding the role of the signal transducer and activator of transcription (STAT) family of proteins in pancreatic tumors. STAT proteins, particularly STAT3, play important roles in pancreatic cancer, especially pancreatic ductal adenocarcinoma (PDAC), which is the most prevalent histotype. The role of STAT3 across a continuum of molecular processes, such as PDAC tumorigenesis and progression, immune escape, drug resistance and stemness, and modulation of the tumor microenvironment (TME), are only a tip of the iceberg. In some ways, the role of STAT3 in PDAC may hold greater importance than that of oncogenic Kirsten rat sarcoma virus (KRAS). This makes STAT3 a highly attractive target for developing targeted therapies for the treatment of pancreatic cancer. In this review, the current knowledge of STAT3 in pancreatic cancer has been summarized, particularly relating to STAT3 activation in cancer cells, cells of the TME, and the state of targeting STAT3 in pre-clinical and clinical trials of PDAC.

## Introduction

Although less common than other cancers, pancreatic ductal adenocarcinoma (PDAC), which represents approximately 90% of cases, is typically diagnosed at a late stage where patient survival is less than 10% [1–3]. Moreover, while less common, pancreatic cancer deaths are expected to rise becoming second only to lung cancer by 2030 [4]. Considering the impact, there is a need to improve our understanding of the disease biology to develop novel therapeutic strategies.

The signal transducer and activator of transcription (STAT) proteins are an important group of transcription factors that serve key functions in the origins, growth, and survival of PDAC [5, 6]. As such, each STAT provides unique insight into these processes, as well as a useful perspective in the context of therapeutics. Understanding the role of STATs in PDAC may provide a framework for future drug development, due to their involvement in cancer cell behavior. In this review, the current literature has been assessed and reviewed, to provide an overview of the current state of understanding regarding the role of the STAT family of proteins in PDAC, with an emphasis on STAT3. Specifically, we explore its role in cancer cells and other cells of the tumor microenvironment (TME), and the crosstalk that takes place to promote tumor progression.

Our current understanding indicates that STAT3 is aberrantly activated in many solid tumors, including PDAC, because of autocrine and paracrine mediated induction by growth factors, and inflammatory cytokines. This is unique from liquid tumors such as leukemia where 30–40% of patients present with an activating mutation in STAT3 [7]. The aberrant activation of STAT3 supports and promotes tumor growth and development through a multitude of processes, which include the promotion of cell proliferation, angiogenesis, metastasis, immune suppression, and stemness. Through these processes, STAT3 acts as a major driving force in PDAC tumorigenesis and progression. For instance, interleukin 6 (IL-6) and STAT3 play critical roles in the promotion of metastasis [8, 9]. Pancreatic stellate cells (PSCs) secrete IL-6 which then activates STAT3 signaling and promotes PDAC progression [9]. In our own studies, we have observed that prolactin (PRL) hormone induces STAT3 activation to increase Panc-1 and MiaPaca2 PDAC cell migration and tumor growth [10]. STAT3 also contributes to the manipulation of the TME. By directly regulating programmed death ligand-1 (*PD-L1*) gene expression in cancer cells, STAT3 plays a vital role in the generation of immune cold pancreatic tumors [11, 12]. Moreover, by regulating tumor metabolism and the specialization of cancer-associated fibroblasts (CAFs), STAT3 can promote a microenvironment which supports tumor growth, development, and progression. This briefly illustrates the broad impact of STAT3 in pancreatic cancer. In this way, disrupting the functions of STAT3 may serve as a key interest in the ongoing battle against PDAC and other malignancies.

## STAT structure and pathway activation

**Figure 1:**
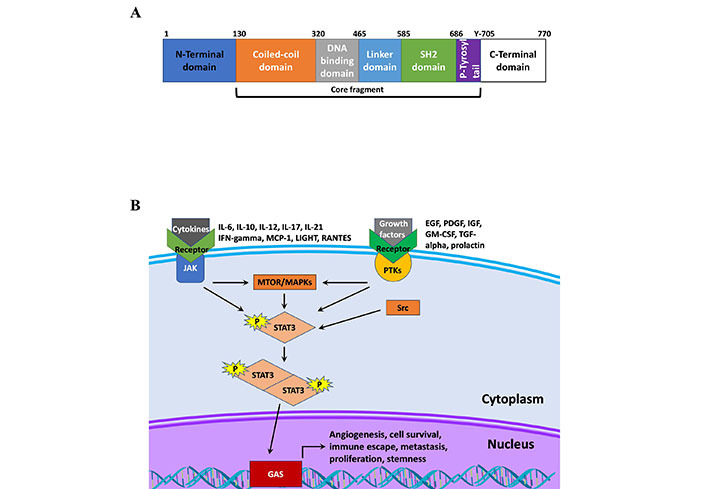
STAT structure and signaling. A. Schematic of STAT3 protein structure; B. schematic of STAT3 signaling. IFN-gamma: interferon-gamma; MCP-1: monocyte chemoattractant protein-1; LIGHT: tumor necrosis factor superfamily member 14; RANTES: chemokine ligand 5; IGF: insulin-like growth factor; GM-CSF: granulocyte-macrophage colony-stimulating factor; TGF-alpha: transforming growth factor alpha; PTKs: protein tyrosine kinases; MTOR: mammalian target of rapamycin; MAPKs: mitogen-activated protein kinases; Src: proto-oncogene tyrosine-protein kinase Src

Functionally, the STAT protein family behave as transcription factors, regulating the expression genes that affect tumor development, progression, and therapy response [13]. Currently, there are seven members in the STAT protein family, STAT1, STAT2, STAT3, STAT4, STAT5a, STAT5b, and STAT6 [14, 15]. The structure of STAT proteins follows a simple pattern conserved across the different members of the STAT family. As shown in Figure 1, all STAT proteins have six conserved domains. The proteins encode an N-terminal domain followed by a coiled-coiled domain, which is critical domain for activation by epidermal growth factor (EGF) or IL-6 [16]. Following this is the DNA binding domain, which has been shown to not only bind DNA at the gamma-activated sequence (GAS), but also other factors such as nuclear factor kappa B (NF-κB) [17]. The protein also codes for a linker domain followed by a Src homology 2 (SH2) domain, which is how STAT proteins are recruited to receptors, and a C-terminal domain [14]. Further details on how each of these domains serve a unique purpose and contributes to the diversity of functions performed by STAT family members in addition to exploring pathway activation is presented by Awasthi et al. [14]. Briefly, STAT proteins are canonically activated by receptor-associated Janus kinases (JAK) following receptor binding to cognate ligands. This leads to phosphorylation of STAT proteins at various tyrosine residues including in the C-terminal domain, and subsequent dimerization that promotes nuclear translocation and DNA binding [14]. Receptors for growth factors and cytokines play a key role in the phosphorylation of STAT3 through the recruitment and activation of JAK proteins, which then directly activate STAT3 [18]. The regulation of gene expression by cytokines and growth factors, such as IL-6, IL-10, IL-23, IL-17, fibroblast growth factor 2 (FGF2), and vascular endothelial growth factor (VEGF) implicates STAT3 as a promoter of cell proliferation and inflammation, stemness, metastasis and chemoresistance (Table 1) [10, 19, 20].

**Table 1:** STAT3 functions and its activators

Function	Activator	Target genes	References
Angiogenesis	IL-6, VEGF, FGF, IL-17	*PDGF*, *VEGF*, *TNF-α*, *TGF-β*, *c-Jun*	[6, 21–26]
Cell proliferation	EGF, IL-6, LDL, PRL	*c-Myc*, *cyclin-D1*	[20, 27–29]
Immune escape	IL-6, IL-10, CXCL12, EGF, IL-22	*IL-6*, *IL-10*, *PD-L1*, arginase 1	[6, 27, 28, 30, 31]
Metastasis	IL-6, IL-28, PRL, CXCL12, LDL	*Snail*, *slug*, *MMPs*, *TWIST*, *ZEB1*	[6, 20, 29, 32]
Survival	IL-6, LDL, PRL, FGF	*Bcl-XL*, *Mcl-1*, survivin	[29, 33, 34]
Stemness	IL-22, PRL, IL-6	*Sox2*, Nanog, *c-Myc*	[20, 32, 35, 36]

LDL: low-density lipoprotein; CXCL12: CXC chemokine (CXC) ligand 12; c-Jun: Jun proto-oncogene; c-Myc: Myc proto-oncogene; MMPs: matrix metalloproteinases; TWIST: class A basic helix-loop-helix protein 38; ZEB1: zinc finger E-box-binding homeobox 1; Bcl-XL: B-cell lymphoma-extra large; Mcl-1: induced myeloid leukemia cell differentiation protein; Sox2: SRY-box 2

These stimuli can also activate Sp1-transcription factor (Sp1), which can cooperate with STAT3 to promote transcription of target genes. Sp1 and STAT3 have distinct target genes, as well as genes that overlap and require cooperative transcriptional regulation [37]. In a study by Gibadulinova et al. [38], the authors demonstrated the in the promoter of S100P, there were adjacent STAT3 and Sp1 binding sites which were critical for gene transcription. In addition to functioning in a cooperative manner via binding to promoter regions within proximity, STAT3 and Sp1 also interact to modulate transcription of target genes, both synergistically and antagonistically [37]. This suggests that many genes controlled by these factors may also require dual inhibition of both STAT3 and Sp1 to effectively inhibit transcription. In pancreatic cancer, Sp1 and STAT3 for example, regulate *VEGF* and beta-FGF (*bFGF*) gene expression to promote angiogenesis, metastasis, and tumor cell proliferation [37]. Given the overlap, further study regarding the crosstalk between Sp1 and STAT3 is necessary, particularly in pancreatic cancer.

## Role of STAT3 in PDAC

### Tumorigenesis

There is considerable evidence demonstrating the importance of STAT3 in cellular transformation and tumor initiation of PDAC. The hyper-activated state of STAT3 is, in part, due to the presence of oncogenic Kirsten rat sarcoma virus (KRAS) mutations, which account for approximately 90% of PDAC cases [39]. The activation of oncogenic KRAS signaling requires stimulation by upstream factors, and thereby promote aberrant activation of STAT3. In this way, understanding the secretome of the TME is critical to developing novel therapies and improving our understanding of PDAC biology.

As it pertains to cellular transformation and tumorigenesis, STAT3 is critical for the development of pre-malignant pancreatic intraepithelial neoplasia (PanIN) and ultimately progression towards PDAC. This was shown in an elegant study performed by Corcoran et al. [40]. The authors observed increased STAT3 phosphorylation at multiple stages of PDAC development, including the earliest PanIN lesions in KC (Pdx1-Cre; LSL-KRAS^G12D^) mouse models [40]. Normal pancreatic tissue, by contrast, had undetectable levels of phosphorylated STAT3 (pSTAT3), suggesting an important role for STAT3 in tumorigenesis and progression. Moreover, is a second study, the authors crossed KC mice with mice containing a conditional knockout (KO) allele for STAT3. STAT3 KO mice exhibited near normal pancreatic histology, demonstrating the critical role of STAT3 in oncogenic KRAS pancreatic cancers [40].

The importance of STAT3 in PDAC tumorigenesis was further confirmed in a study by Loncle et al. [25], where the authors demonstrated that IL-17 induced acinar-to-ductal metaplasia (ADM) and PanIN formation in a STAT3 dependent manner [25]. Specifically, the authors determined that IL-17 induced the expression of regenerating islet-derived protein 3β (REG3β), which in turn promoted cell growth and anti-apoptotic signals [25]. Moreover, REG3 induced the expression of IL-10 and TGF-β via the CXCL12/CXC chemokine receptor 4 (CXCR4) axis, resulting in alternatively activated, or M2 polarized macrophages in co-culture experiments [25]. These data demonstrate that STAT3 contributes to PDAC tumorigenesis and progression, implying a strong rationale for use of STAT3 inhibitors for early intervention strategies of pancreatic cancer.

### Proliferation and metastasis

The regulation of multiple key functions within the context of PDAC falls within the functions of STAT3. Both IL-28 receptor, alpha subunit (IL-28RA) and glutathione S-transferase Mu 3 (GSTM3) play a role in the regulation of STAT3 activity, which when overactive, plays a role in cell proliferation, survival, angiogenesis, and metastasis as demonstrated by a reduction of VEGF, when STAT3 is inhibited [6, 41]. Moreover, STAT3, in part, functions as a driver of PDAC growth. When PDAC cells were treated with the EGF receptor (EGFR) inhibitor, AG490, STAT3 activation was inhibited which caused a decrease in cyclin protein expression [6]. The decreased expression indicates that STAT3 plays a critical role in the regulation of the cell cycle and proliferation. In this way, STAT3 serves as a potential therapeutic target, especially in malignancies such as PDAC. Furthermore, using xenograft cancer models *in vivo* indicated that inhibiting STAT3 with panaxadiol limited the progression of pancreatic cancer [42]. This study also indicates that the use of panaxadiol inhibits cellular processes such as proliferation, migration, and metastasis in Panc-1 and Patu8988 cell lines. Observing the induction of apoptosis in pancreatic cell lines and its strong affinity to STAT3, panaxadiol offers useful insights into the role of STAT3 in cancer proliferation and metastasis through its inhibition.

STAT3 performs important functions, many of which promote an aggressive phenotype for malignant tissues. In normal skin tissues, STAT3 promotes wound healing through cell migration, which cancer cells take advantage of as seen with the migration of ovarian cancer cells [43, 44]. In the context of PDAC, IL-6 and STAT3 each play a role in the promotion of metastasis [8, 9]. Specifically, PSCs secrete IL-6 which activates STAT3 signaling in benign neoplasms and malignant cells, promoting PDAC progression [9]. Inhibition of this crosstalk by using an IL-6 neutralizing antibody, decreased PDAC cell invasion, and colony formation, and STAT3 activation [9]. Certain tumor protein P53 (P53) mutations also promote the migration and invasion of PDAC by promoting the hyperactivation of STAT3 [45]. Mutant P53^R248W^ protein is stabilized by heat shock protein 90 (HSP90) leading to gain-of-function activity that leads to a hyperactivation state of STAT3, and in turn proliferation and migration. Treatment with HSP90 inhibitors, such as onalespib and ganetespib, causes degradation of mutant P53, thereby reducing STAT3 phosphorylation.

Another method of STAT3 activation relies on the PRL receptor (PRLR) as observed in hormone-dependent cancers, primarily breast cancer, though an observed increase in PRL expression also persists in PDAC [10, 20, 46]. In our previous study, we demonstrated that PRL binds to the extracellular domain of PRLR which promotes an association with JAK2, thus inducing an activation cascade of downstream pathways, such as STAT3 and STAT5 [20]. This increased cellular proliferation, cell motility and stemness of Panc-1 and MiaPaCa-2 PDAC cell lines through STAT3 mediated upregulation of the oncogene, Myc. Moreover, we determined that by inhibiting PRLR induced activation of STAT3 using pharmacologic and genetic approaches, these cellular phenotypes were significantly impaired. As such, PRLR presents an interesting opportunity for the development of therapeutics which target upstream factors of the STAT3 pathway for the treatment of PDAC.

Other health factors influence the function of STAT3 as alternative mechanisms of activation. In PDACs, LDL cholesterol activates JAK1 and JAK2 and non-receptor tyrosine kinases like Src, which promotes cell proliferation and survival by the subsequent activation of STAT3 [29]. Moreover, LDL mediated activation of STAT3 enhances PDAC cell migration and invasion. Treatment of these cancer cells with simvastatin reduced LDL-induced proliferation, cell mobility, and STAT3 activation [18]. This implicates LDL cholesterol in the development and progression of PDAC and indicates that high levels of LDL cholesterol pose an increased risk of PDAC in patients.

### Stemness phenotype

There is evidence that a small population of cells within a tumor have an increased capacity to resist therapy and cause tumor recurrence. These cancer stem cells (CSCs) often promote chemoresistance and the recurrence of the disease since they avoid common therapies targeting rapidly dividing cells. These CSCs aren’t necessarily transformed stem cells, but rather malignant cells that have increased ATP-binding cassette (ABC) transporter expression, and DNA repair and reactive oxygen species (ROS) scavenging capabilities [47–49]. Moreover, these CSCs exhibit self-renewal and anchorage independent growth properties [50–53]. As a component of regulating the self-renewal of stem cells, as well as cell survival and inflammation, STAT3 serves a significant role in pancreatic cancer [40]. Recently He et al. [35], determined that IL-22/IL-22RA1 signaling axis contributes to tumor heterogeneity and increased percentage of stemlike cells within the tumor [35]. Accordingly, STAT3 was indispensable for IL-22 induced stemness in pancreatic cancer cells, demonstrating the importance of STAT3 in the pathway. We have also observed that STAT3 enhanced spheroid formation, a surrogate for stemness, in PDAC cells through a PRL: PRLR signaling axis [10]. This may be in part due to increased expression of the STAT3 target gene, Myc. Inhibition of PRLR prevented PRL induced STAT3 phosphorylation and attenuated spheroid formation and induction of stemness genes [10]. As such, STAT3 provides an opportunity for the development of therapeutics that target CSCs.

### Metabolism

A key difference between normal and cancer cells is cellular metabolism. As major components in the regulation of cytokine induced survival and proliferation, STAT3 and STAT5 play an important function in tumor growth and survival through the regulation of metabolic processes, such as glycolysis and oxidative phosphorylation [54–57]. In low glucose conditions, nuclear and total STAT3 is decreased, and upon ectopic overexpression of STAT3, cells exhibit increased glucose consumption, indication that aerobic glycolysis is impacted by STAT3 signaling [56]. In another study by Li et al. [55], STAT3 overexpression led to the direct increases in glucose consumption and formation of lactate. This was accompanied by increased expression of hexokinase 2 mRNA and protein, suggesting the direct regulation of glycolytic genes by STAT3 [55]. In the context of PDAC, Valle et al. [57], demonstrated that when galactose was substituted for glucose, PDAC cells utilize oxidative phosphorylation to a greater extent [57]. This caused PDAC cells to become more tumorigenic by enriching for CSC populations and promoting chemoresistance and immune escape [57]. This was associated with noted upregulation in IL-6/STAT3 signaling by ribonucleic acid sequencing (RNA-seq), though validation of the direct role of STAT3 was not tested. Additionally, STAT1 and STAT6 also play a role in metabolism as STAT1, STAT3, STAT5, and STAT6 each play a role in the creation of a dynamic metabolism within cancer cells [58]. STAT3, in particular, supports cancer cell survival in a metabolically hostile environment by promoting an increase in glycolysis through the Warburg effect, which increases proliferation of these cells quickly draining the area around a tumor of vital nutrients and resources [59]. Surrounding cells become starved, while the cells within the tumor continue to thrive, allowing cancer cells to out compete. In this way, the STAT family of proteins provide PDAC tumors with incredible metabolic versatility and adaptability while also influencing other cells within the TME.

## Role of STATs in the TME

A recent topic of study, the TME, has gained much interest due to increasing evidence of the significance it has on tumor progression. It is a multicellular network composed of immune cells, fibroblasts, adipocytes, and symbiotic organisms. The STAT family of proteins plays key roles in interactions between cancer cells and the TME, often serving the function of mounting an immune response. In the context of PDAC, genes regulated by STAT3 often fall into the category of cytokines, growth factors, or angiogenic factors that in turn activate STAT3. An example of this positive feedback loop is seen with IL-6 and VEGF [60, 61]. This feed-forward loop also allows for the perpetual activation of STAT3 and continuous expression of these pro-tumor genes. Furthermore, this promotes the growth and development of PDAC tumors and contributes to the creation and maintenance of a pro-tumor TME [61].

### Immune cells

STAT3 regulates the communication between cancer cells and immune cells and influences immune escape of tumors [62, 63]. That is, STAT3 drives the immunologically cold state of PDAC tumors. For example, PD-L1 plays an important role in PDAC tumor evasion of the immune system [64]. Under normal circumstances, PD-L1 regulates immune evasion preventing autoimmunity, a function which PDAC tumors exploit [65]. Histone deacetylase 3 (HDAC3) regulates the expression of PD-L1 through the STAT3 pathway as demonstrated by the observed decrease in both PD-L1 and phosphorylated STAT3 in the absence of HDAC3 [66]. This illustrates the importance of STAT3 in the regulation of tumor evasion of the immune system.

The role of STAT3 extends beyond the basic communication between tumors and the TME. Cancer cells require greater resources and nutrients than surrounding cells to sustain their increased proliferation rate. As mentioned earlier, this is regulated in part, by STAT3. The changes in cellular metabolism by cancer cells can also influence the anti-tumor immune responses. The increased nutrient demands by cancer cells can cause oxidative stress on natural killer (NK) cells, which in turn reduces their anti-tumor capabilities [59, 63]. As such, the influence of STAT3 on the TME favors a pro-tumor environment that promotes immune escape. In this way, the manipulation of STAT3 may allow for reactivation of the immune system in patients, thus making STAT3 a potential target in conjunction with immunotherapy. Additionally, the anti-tumor properties of NK cells return upon the activation of the nuclear factor erythroid 2-related factor 2 (Nrf2) antioxidant pathway, thus overriding the negative impacts of STAT3 activation on the immune cells [59]. Using this pathway in conjunction with current immunotherapies may allow for the development of improved treatments for PDAC and other cancers.

### Fibroblasts

STAT3 also impacts other components of the TME such as the phenotype of fibroblasts, especially those associated with malignant tumors. As such, IL-1 expression in conjunction with the activation of the JAK/STAT pathway, promotes the heterogeneity and inflammation of CAFs which may serve multiple functions [67]. Specifically, the authors determined that two types of fibroblasts exist within PDAC tumors. These two populations of PDAC CAF cells express different markers, critical for their specific functions, with inflammatory CAFs (iCAFs) expressing markers of inflammation such as IL-6 and leukemia inhibitory factor (LIF), and myofibroblastic CAFs (myCAFs) expressing markers of myofibroblasts such as alpha smooth muscle actin (αSMA) [67]. Tumor proximity also segregates these two populations of fibroblast cells, with myCAFs maintaining adjacent positioning with the tumor while the location of iCAFs remains in the stroma further away, though the driving factor for this differential expression remains a mystery [67]. Nevertheless, the secretion of IL-6 by iCAFs can perpetuate the feed-forward loop described above. Interestingly, the authors also demonstrate that TGF-β inhibits IL-1R1 on CAFs, promoting differentiation into a myofibroblasts [67]. This generates a different TME, that is characterized by increased fibrosis. This acts as a double-edged sword, where on one hand the increased fibrosis creates a physical barrier that impairs cancer cell migration, but on the other hand also impedes immune cell infiltration and chemotherapy delivery to the tumor. The authors further demonstrate the role of STAT3, as decreased expression of the iCAF marker genes was observed following STAT3 knockdown [67].

These findings were further supported by a study published by Velez-Delgado et al., where the authors demonstrated that PDAC tumors harboring a KRAS^G12D^ mutation had increased secretion of inflammatory cytokines, such as CXCL1, IL-6, IL-33, and serum amyloid A (Saa3) that lead to activation of STAT3 in CAFs within the TME [68]. Specifically, the authors utilized a mouse model of pancreatic cancer with inducible and reversible expression of the KRAS^G12D^ mutation. The authors performed single-cell RNA-seq and mass-cytometry to evaluate the role of mutant KRAS in the TME. These studies demonstrated that oncogenic KRAS signaling within the epithelial tumor cells created an inflammatory secretome that reprogrammed normal fibroblasts towards a tumor promoting phenotype [68].

Collectively, these studies demonstrate that the CAF phenotype in PDAC relies, at least in part, on STAT3 activation. Moreover, the aberrant activation of STAT3 provides direct benefits within the tumor cell itself, and the added benefits of fibroblast reprogramming in the TME to support tumor growth and survival. As such, it will be important to consider the stromal heterogeneity surrounding PDAC for future therapy developments.

### Targeting STAT3 in PDAC

The critical role of STAT3 across a broad scope of malignancies has encouraged the development of selective inhibitors for therapeutic and research purposes. Multiple clinical trials continue investigating treatments for PDAC though as the implication of STAT proteins driving the progression of PDAC continues, the need for clinical trials investigating the effectiveness of novel therapies rises. Several clinical trials far have focused on targeting STAT3, yet only two have incorporated pancreatic cancer in the scope of study, to date (Table 2).

**Table 2:** STAT3 inhibitors in clinical trials

**Phase**	**Tumor type**	**Drug**	**Status**	**Trial ID**
II	Colon, lung, pancreatic	AZD9150 and MEDI4736	Ongoing	NCT02983578
I	Head and neck	OPB-51602	Terminated due to toxic metabolites of drug	NCT02058017
II	All advanced solid tumors	DSP-0337	Terminated due to alternative development strategy	NCT03416816
II	Leukemia	Flavopiridol	Terminated due to adverse effects	NCT00098371
III	Pancreatic	Napabucasin	Terminated due to lack of OS improvement	NCT02993731

OS: overall survival

A phase II clinical trial with intravenous administration of an anti-sense STAT3 molecule, AZD9150 in combination with an anti-PD-L1 agent, MEDI4736 is currently underway in patients with advanced PDAC. A total of fifty-three patients with either PDAC, as well as those with non-small cell lung cancer (NSCLC), and mismatch repair deficient colorectal cancer. The current anticipated completion date for this trial is April 30, 2024 (NCT02983578). Once complete, this clinical trial may provide unique insight into the feasibility of targeting STAT3 and therefore, warrants revisiting at the time of completion.

The phase III “CanStem111P” clinical trial that was initiated in 2017 was recently terminated due to a lack of OS improvements. This open-label trial across 165 study sites world-wide sought to evaluate the efficacy and safety of combining the STAT3 inhibitor napabucasin with nab-paclitaxel and gemcitabine in patients with metastatic PDAC (NCT02993731). According the Bekaii-Saab et al. [69], 565 patients were randomized into a napabucasin treatment arm and 569 received control (nab-paclitaxel and gemcitabine). Median OS in the napabucasin treatment arm was 11.4 months *versus* 11.7 months in the control arm. Moreover, no significant difference in OS was observed between biomarker positive subgroups (pSTAT3 positive) [69].

Despite the potential of STAT3 inhibitors in PDAC based on preclinical studies, many trials investigating STAT3 inhibitors in many cancers have failed. This unfortunately creates challenges for evaluating these compounds in pancreatic cancer. One clinical trial studied dosing of OPB-51602, an inhibitor of STAT3 phosphorylation, in solid nasopharyngeal carcinoma tumors. This trial failed due to the intolerable nature of lactic and metabolic acidosis of OPB-51602 (NCT02058017). The consideration of toxic metabolites of a potential drug remains an important factor when determining the viability of such compounds.

Another terminated clinical trial focused on DSP-0337 in patients with solid tumors and ceased activity due to “alternate development strategy” (NCT03416816). Another phase II clinical trial sponsored by the National Cancer Institute investigating the use of flavopiridol in patients suffering from chronic lymphocytic leukemia or prolymphocytic leukemia failed due to serious adverse events (NCT00098371) [70]. While these clinical trials discovered unique issues causing their termination, other trials showed difficulty in recruiting patients. In this way, many clinical trials regarding STAT3 inhibitors in the context of cancer have failed, necessitating the need for new strategies of targeting STAT3 signaling.

Since current STAT3 inhibitors have largely failed in the clinical setting, there is a need to develop novel drugs and strategies to target STAT3. Recently, several small molecule inhibitors have been developed and tested in pre-clinical models of pancreatic cancer. In a study by Chen et al. [71], the authors identified a novel small-molecule inhibitor (N4) that blocked STAT3 phosphorylation by interfering with the SH2 domain. This further prevented STAT3 dimerization and suppressed tumor growth and metastasis in mouse models of pancreatic cancer, providing the justification for the small molecule inhibitor as a potential therapeutic for PDAC [71]. In another study by Zheng et al. [72], the authors tested a novel agent, W2014-S, in NSCLC. This compound worked by not allowing STAT3 to dimerize, and as a result suppressed STAT3 signaling [72]. In animal models of NSCLC, including patient-derived xenograft (PDX) tumor xenografts, W2014-S inhibited cancer growth, demonstrating proof of concept in preclinical studies of STAT3 driven NSCLC tumors [72]. Further evaluation in PDAC may be warranted based on these findings.

## Areas of future research

Understanding the various proteins of the STAT family and their independent functions allow for the improvement of the current standard of care for patients with PDAC through the development of novel therapeutics and strategies. Unfortunately, the literature currently available on the STAT family of proteins and their functions in PDAC only scratch the surface regarding this area of study. This necessitates further studies on the roles and functions of the STAT protein family in PDAC.

Since inflammation plays a significant role in tumorigenesis as well as tumor growth and development, understanding the generation and maintenance of myCAFs and iCAF populations in relation to the JAK/STAT pathway may provide opportunities for developing therapies with higher efficacy for PDAC. Moreover, strategically targeting the JAK/STAT pathway also provides opportunities for manipulation of the TME that may prevent the development and progression of tumors at early or pre-cancerous stages of the disease.

Furthermore, understanding how the native human microbiome impacts the expression of STAT3 remains understudied. Evidence indicates that bacterial metabolites such as indole-3-acetic acid (3-IAA) influence the effectiveness of chemotherapies by manipulating the susceptibility of PDAC [73]. This study indicates an important relationship between the host response to therapy, their microbiome, as well as host gene expression. As such, interactions between bacterial metabolites, the expression of STAT proteins in PDAC tumors, and STAT protein expression in the TME remain an overlooked, yet valuable area of study. Since multiple proteins within the STAT family function in the mounting of an immune response for bacterial and viral infections, interactions between the expression of these STAT proteins and the native human microbiome deserves further investigation in the future.

Despite the failure of many drugs targeting STAT3 in the clinical setting, there remain approaches to modulate STAT3 activation, albeit indirectly, that may provide effective alternate therapeutic strategies. For example, as with the PRLR mediated activation of STAT3, targeting this upstream regulator of the pathway provides a promising outlook on treating patients with various cancers including PDAC. Although targeting PRLR with chemotherapy may cause complications with pregnancies and lactation, these factors pose little risk regarding most pancreatic cancer patients, due to the average age of diagnosis occurring over the age of 71 [74]. Furthermore, other upstream components of STAT3 activation may serve as vital targets for novel drugs and therapies that limit the functions of STAT3 without direct interactions.

Expanding the scope of potential therapeutic targets beyond STAT3 allows for the initiation of clinical trials investigating the efficacy of therapies on other critical transcription factors in cells of the PDAC tumor. As such, STAT proteins such as STAT5 and STAT6 warrant further studies in this regard. As the need grows for an improvement in the current standard of care for patients with PDAC, future studies involving the STAT family of proteins should promote the initiation and progression of clinical trials along with the expansion of understanding regarding the functions each of these proteins serve in different malignancies. In this way, investigations into other targets in the STAT family of proteins such as STAT1, STAT2, STAT4, STAT5, and STAT6 should continue so that standard of care for patients with PDAC may improve through a better understanding of these factors.

## Conclusions

As new evidence unveils the new roles of STAT family proteins in PDAC, opportunities arise for the improvement of the current standard of care for patients with the PDAC malignancy. As such, placing an emphasis on the current understanding and the gaps within, are critical to improving survival prospects for patients diagnosed with pancreatic cancer.

Each protein in the STAT family serves essential functions in the pancreas and surrounding tissues, often promoting an environment that supports the growth and development of PDAC tumors. As such, determining the unique functions of each STAT protein in PDAC allows for the addition of critical information that remains absent in the current understanding of these transcription factors and their role in PDAC which continuously inhibits the development of therapeutics and the progression of clinical trials.

Further consideration into the biological functions of STAT2 may also yield a path for novel therapeutics and preventative measures through its regulation of pancreatic inflammation. As such, therapeutic targets may allow for therapies that bolster expression and functionality for the desired effect of treating PDAC and reducing future cases.

Although the current understanding of the role each STAT protein plays in PDAC remains scattered, studies continue the push for bridging the chasms in the field so that achieving a complete understanding of the subject matter remains possible. The challenges presented by these gaps in understanding form a motivational environment encouraging the continued expansion of published knowledge on the topic by investigators. Since STAT3 and STAT5 currently remain the most thoroughly studied, there exists a self-evident need for additional research on the other STAT proteins so that the development of novel therapies and preventative measures continues.

The roles played by the STAT family proteins in the context of PDAC remain critical for the function and survival of pancreatic tumors making the need for additional studies and clinical trials important for the furthering of understanding and standard of care improvement.

## Abbreviations

CAFs: cancer-associated fibroblasts
CSCs: cancer stem cells
CXCL12: CXC chemokine ligand 12
EGF: epidermal growth factor
FGF2: fibroblast growth factor 2
iCAFs: inflammatory cancer-associated fibroblasts
IL-28RA: interleukin 28 receptor, alpha subunit
IL-6: interleukin 6
JAK: Janus kinases
KRAS: Kirsten rat sarcoma virus
LDL: low-density lipoprotein
myCAFs: myofibroblastic cancer-associated fibroblasts
NSCLC: non-small cell lung cancer
OS: overall survival
P53: tumor protein P53
PanIN: pancreatic intraepithelial neoplasia
PDAC: pancreatic ductal adenocarcinoma
PD-L1: programmed death ligand-1
PRL: prolactin
PRLR: prolactin receptor
Sp1: Sp1-transcription factor
Src: proto-oncogene tyrosine-protein kinase Src
STAT: signal transducer and activator of transcription
TGF-alpha: transforming growth factor alpha
TME: tumor microenvironment
VEGF: vascular endothelial growth factor

## Declarations

### Copyright

© The Author(s) 2024.
